# Amoebic Liver Abscesses in Returning Travelers: Lessons to Keep in Mind From a Nonendemic Area

**DOI:** 10.1155/crdi/9933233

**Published:** 2025-08-21

**Authors:** Anna Barbiero, Sasha Trevisan, Tommaso Manciulli, Jessica Mencarini, Annalisa Cavallo, Costanza Fiorelli, Alessandro Bartoloni, Michele Spinicci, Lorenzo Zammarchi

**Affiliations:** ^1^Experimental and Clinical Medicine Department, University of Florence, Florence, Italy; ^2^Infectious and Tropical Diseases Unit, Careggi University Hospital, Florence, Italy

**Keywords:** amebiasis, amebic liver abscess, Colombia, Italy, travel medicine

## Abstract

Despite being relatively common in endemic settings, amoebic liver abscesses are rarely diagnosed in high-resource countries, where they are usually imported by migrants or travelers to endemic areas. Between 2010 and 2024, we observed two cases of amoebic liver abscess that were successfully managed in a tertiary-care center in Italy. In both cases, the infection was contracted in Colombia. Given the high morbidity and mortality associated with nontreated amoebic liver abscesses, this report aims at underlying the importance of travel history and of considering amoebic liver abscess among the differential diagnoses for people coming from endemic countries with compatible clinical presentations. Prompt diagnosis and treatment are of paramount importance for favorable clinical outcomes.

## 1. Introduction

Intestinal amebiasis (IA) is a widespread infection caused by the protozoan *Entamoeba* spp., comprising *Entamoeba histolytica* (*Eh*), *E. dispar* (*Ed*)*, E. moshkovskii (Em),* and *E. bangladeshi* (*Eb*). IA occurs mostly in low–middle-income countries in Asia, Africa, and Latin America and is acquired by ingesting food or water contaminated by feces containing parasitic cysts. Sexual transmission by fecal–oral contact has also been described. While infections by *non-histolytica* species are usually nonpathogenic, *Eh* can cause colitis and dysentery, even if most cases (about 90%) are asymptomatic. Invasive amebiasis is associated with a high morbidity and mortality rate without proper treatment, representing the third parasitic cause of death worldwide, after malaria and schistosomiasis, with 100,000 deaths recorded annually [1]. Amoebic liver abscess (ALA) is the most common extraintestinal manifestation of *Eh* infection [2]. More rarely, *Ed* can also be a cause of ALA [3]. ALAs typically present with fever and right upper quadrant pain, in some cases accompanied by cough, sweating, malaise, and weight loss, whereas jaundice occurs in less than 10% of cases. While diarrhea is concurrent in a minority of cases, a proportion of patients report a history of dysentery within the previous few months [4]. The most severe evolutions of ALAs include rupture and consequent involvement of the peritoneal and/or pleural cavity, the latter being about four times more frequent than the former, evolving into peritonitis and/or pleural empyema, respectively. ALAs, especially those located in the left hepatic lobe, can also rupture into the pericardium, resulting in pericardial effusion, acute pericarditis, myocarditis, pericardial abscess, congestive cardiac failure, pericardial tamponade, or constrictive pericarditis, with high associated mortality [5]. We present two cases of ALA seen at a tertiary care center in Italy.

## 2. Case 1

**Figure 1:**
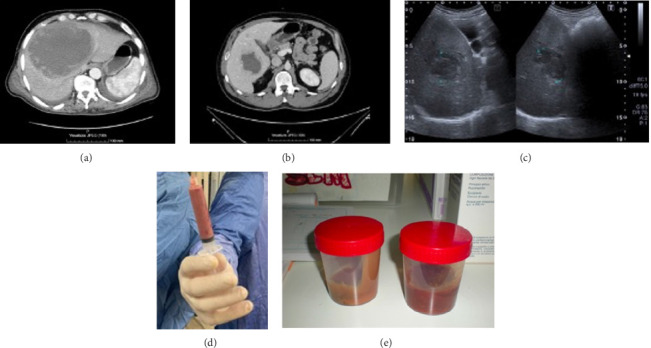
Appearance of the amoebic abscess of Case 1 (a) and Case 2 (b) on the CT scan. (c) Appearance of the amoebic abscess of Case 2 on ultrasound; “anchovy paste” appearance of the material drained from Case 1 (e) and Case 2 (d) abscesses.

On February 17th, 2010, a 68-year-old Italian man, frequently travelling to Colombia, was admitted to a hospital in Bogotá for fever and right pleural effusion, which was treated with fluoroquinolone on the suspicion of a pulmonary infection. He reported having recurrent episodes of unspecified colitis in the previous years. During hospitalization, he remained febrile, diarrheic, and asthenic. He came back to Italy after self-discharge and was admitted at the Infectious and Tropical Diseases Unit of Careggi University Hospital, Florence, on February 22nd, 2010. On physical exam, he was icteric and had abdomen tenderness. Blood exams showed mild leukocytosis (WBC 10.800 × 10^9^/L, normal values 4.00–10.00 × 10^9^/L), creatinine 1.97 mg/dL, AST 247 U/L (n.v. 5–40 U/L), ALT 321 U/L (n.v. 5–40 U/L), and total bilirubin 4.62 mg/dL (n.v. 0.3–1.00 md/dL). A chest X-ray showed bilateral pleural effusion and a left basal infiltrate. Abdominal ultrasound showed a hypoechoic 11 cm solid hepatic lesion, suspected of ALA. The patient initially started antibiotic therapy for pneumonia (piperacillin/tazobactam plus azithromycin) with the addition of metronidazole and paromomycin due to ALA suspicion. Contrast-enhanced CT confirmed the ultrasound findings, showing a 15.3 × 12.8 cm cystic lesion occupying the IV–VIII hepatic segment with a peripheral enhancing rim, compatible with the diagnostic hypothesis of amoebic abscess (Figure 1(a)); “anchovy paste” material was drained from the hepatic lesion (Figure 1(e)). A qualitative immunochromatographic test for *Ed/Eh* antigen performed on the drained material resulted positive. Serology against *Eh* (immunofluorescent assay, IFA) was positive (antibody titer 1:800, negative if < 1:50), as well as qualitative *Ed/Eh* stool antigen research. Direct stool microscopy was negative. Imaging follow-up documented progressive resolution of the ALA after therapy with metronidazole and paromomycin for 15 days. Despite pleural fluid not being drained at the time of diagnosis, the pleural effusion was solved on subsequent radiological controls. As regarding transaminases and bilirubin elevation reported at the time of diagnosis, these findings were normalized within two weeks after treatment initiation.

## 3. Case 2

In April 2024, a 46-year-old Colombian man was admitted to the Infectious and Tropical Diseases Department of Careggi University Hospital, Florence. While travelling around Europe for tourism, he had developed abdominal pain and loose stools, followed by fever, nausea, and abdominal pain. Blood exams showed leukocytosis (WBC 16.800 × 10^9^/L), anemia, and increased procalcitonin (1.33 ng/mL, n.v. < 0.5 ng/mL) and C-reactive protein (309 mg/L, n.v. < 5 mg/L), with normal transaminases and bilirubin levels. Abdominal ultrasound showed a 52 × 44 mm hypoechoic hepatic lesion, whereas chest X-ray was normal. A contrast-enhanced CT scan showed a hypointense hepatic lesion with a peripheral enhancing rim, which deemed suspect for ALA (Figures 1(b) and 1(c)). Pending confirmation, antibiotic therapy with piperacillin/tazobactam and metronidazole was administered. Direct microscopy on stool was positive for *Eh/Ed* cysts. Stool PCR and *Eh* serology (enzyme-linked immunosorbent assay [ELISA]) also tested positive. The *Eh/Ed* antigenic assay on stools resulted negative. The lesion drainage showed an “anchovy paste,” on which *Eh* PCR and antigen both tested positive (Figure 1(d)). Due to travel-related needs, the patient was discharged with indications to a 10-day course of metronidazole, followed by seven days of paromomycin and clinical and radiological follow-up.

## 4. Discussion and Conclusions

*Eh* infects 10% of the population worldwide, causing 50 million invasive infections and 100,000 deaths every year [4]. Most infections occur in low–middle-income countries, whereas cases observed in high-resource settings are usually imported by migrants and travelers to endemic areas, although rare autochthonous cases in nonendemic areas occur. As in our two examples, men are more frequently affected by ALA, despite equal distribution of IA between sexes [6]. Interestingly, both our cases acquired the infection in Colombia, where a national survey of intestinal parasitic infections between 2012 and 2014 estimated a prevalence of 12.9% for *Eh/Ed/Em* and a 60.3% prevalence for other protozoan intestinal infections. More recent data agree with these findings: a prevalence of 9.1%–20.0% of IA was found in school-aged children in the same country [7, 8]. Hence, travelers should adopt precautions to avoid the infection when visiting Colombia. Moreover, healthcare personnel in nonendemic areas should keep ALA and IA in the differential diagnosis of patients who traveled to endemic countries and present with clinical symptoms compatible with IA or invasive amebiasis, such as diarrhea, fever, and abdominal pain associated with hypointense/hypoechoic hepatic focal lesions. Clinical symptoms for ALA are nonspecific and may mimic many other conditions, especially in the presence of complications (intrapericardiac rupture and intra-abdominal rupture). While the radiological aspect of ALA usually allows to differentiate it from hepatic lesions caused by malignancies or other parasitic infections such as echinococcosis, differentiation from pyogenic abscesses can be more challenging. In these cases, accurate clinical and epidemiological history covers an important role, with ALA being more common in migrants or travelers to endemic areas and pyogenic abscesses being more frequently reported at latitudes where *Eh* is not endemic, frequently in subjects with a recent history of cholecystitis, other abdominal infections, sepsis, and/or immunodepression [9]. It should also be noted that chest abnormalities such as pleural effusion are described in about half of ALA cases as concomitant findings, not necessarily indicating abscess rupture into the pleural cavity [10]. This could be the case of Case 1, in which pleural fluid aspiration and analysis were not performed due to rapid resolution of the pleural effusion and absence of any impact on respiratory dynamics.

Diagnosis of ALA should always be confirmed through serologic or antigenic testing, as well as microscopy and molecular or antigenic assays on stool or liver abscess fluid when possible. However, since amebic colitis is not commonly concomitant to ALA, negative direct tests on stool samples do not exclude the diagnosis if the suspicion is relevant [11]. In the described cases, suspicion of ALA was based on a combination of compatible clinical and radiological presentation combined with a suggestive epidemiological history. Nonetheless, since pyogenic abscess could not be ruled out until microbiological confirmation, both patients were initially administered both large-spectrum antibiotics and specific therapy for ALA, the former being suspended only after microbiological confirmation of ALA. Microbiological assays used in our cases are now widely available and include stool and aspirate antigen detection as well as PCR. Due to widespread exposure in endemic areas, diagnosis in migrants or tourists from high-burden settings cannot rely on serology only. However, serology can be useful for tourists from low-burden areas.

Medical treatment of amebiasis requires the use of an intraluminal agent (in our cases, paromomycin) associated with a tissue-active agent such as metronidazole in invasive cases. Drainage is recommended by experts in ALAs larger than 8–10 cm, in the absence of randomized studies. However, a Cochrane review found no difference in mortality when comparing medical treatment and drainage, even in larger lesions [12]. In our cases, drainage had a therapeutic role in Case 1. We chose to drain Case 2 due to diagnostic needs as the patient was about to go back to his home country, and diagnostic confirmation was needed to establish definitive treatment.

Our cases highlight the importance of accurate epidemiological medical history including recent travels. Since available treatments for ALAs are associated with excellent response and nontreatment with high morbidity and mortality, early diagnosis and prompt treatment are key elements for an optimal clinical management.

## Data Availability

Data sharing is not applicable to this article as no new data were created or analyzed in this study.

## References

[1] Stanley S. L. (2003). Amoebiasis. *The Lancet*.

[2] Haque R., Huston C. D., Hughes M., Houpt E., Petri W. A. (2003). Amebiasis. *New England Journal of Medicine*.

[3] Ximénez C., Cerritos R., Rojas L. (2010). Human Amebiasis: Breaking the Paradigm?. *International Journal of Environmental Research and Public Health*.

[4] Maltz G., Knauer C. M. (1991). Amebic Liver Abscess: A 15-Year Experience. *American Journal of Gastroenterology*.

[5] Adams E. B., MacLeod I. N. (1977). Invasive Amebiasis. I. Amebic Dysentery and Its Complications. *Medicine (Baltimore)*.

[6] Acuna-Soto R., Maguire J. H., Wirth D. F. (2000). Gender Distribution in Asymptomatic and Invasive Amebiasis. *American Journal of Gastroenterology*.

[7] Bryan P. E., Romero M., Sánchez M. (2020). Urban Versus Rural Prevalence of Intestinal Parasites Using Multi-Parallel Qpcr in Colombia. *The American Journal of Tropical Medicine and Hygiene*.

[8] Castañeda S., Acosta C. P., Vasquez L. R., Patiño L. H., Mejía R., Ramírez J. D. (2024). Molecular Detection of Intestinal Parasites in a Rural Community of Colombia: A One Health Approach to Explore Potential Environmental-Zoonotic Transmission. *Zoonoses Public Health*.

[9] Chang C. Y., Radhakrishnan A. P. (2022). Amoebic Liver Abscess. *Revista da Sociedade Brasileira de Medicina Tropical*.

[10] Thorsen S., Rønne-Rasmussen J., Petersen E., Isager H., Seefeldt T., Mathiesen L. (1993). Extra-Intestinal Amebiasis: Clinical Presentation in a Non-Endemic Setting. *Scandinavian Journal of Infectious Diseases*.

[11] Fotedar R., Stark D., Beebe N., Marriott D., Ellis J., Harkness J. (2007). Laboratory Diagnostic Techniques for Entamoeba Species. *Clinical Microbiology Reviews*.

[12] Chavez-Tapia N. C., Hernandez-Calleros J., Tellez-Avila F. I., Torre A., Uribe M. (2009). Image-Guided Percutaneous Procedure Plus Metronidazole Versus Metronidazole Alone for Uncomplicated Amoebic Liver Abscess. *Cochrane Database of Systematic Reviews*.

